# Supplementary material for: Mindfulness-Based Interventions for Undergraduate Nursing Students in a University Setting: A Narrative Review
Source: Healthcare (Basel). 2021 Nov 2;9(11):1493. doi: 10.3390/healthcare9111493 (PMC8621067; doi:10.3390/healthcare9111493)
Supplement: Supplementary file 1 [file healthcare-09-01493-s001.zip › healthcare-1442254-supplementary.pdf]

Table S1. Characteristics of Studies.

Author (year)	Country	Design	Sample size	Participants	Intervention	Intervention period	Outcome Measures	Results
Barbosa et al. (2013)	USA	Quasi-experimental trial	13 (I) 15 (C)	UG nursing Student (n=4) Podiatry students (n=7) Physician assistant (n=5) Physical Therapy (n=7) Occupational Therapy (n=5)	MBSR	Students attended eight weekly classes for 2.5 hours, plus an 8-hour silent day-long retreat during the 6th week	Anxiety [41], Empathy [42] and Burnout [43]	For the intervention group, 85% of students experienced diminished anxiety, from baseline, at both 8 and 11 weeks. For the control group, 13% and 27% of students experienced diminished anxiety at weeks 8 and 11. Findings were statistically significant (P<0.001 and P<0.01, respectively).<br><br>In relation to empathy, a significant difference was observed between

								the experimental and control groups at week 8 (P<0.0096), but this was not sustained at week 11.<br><br>In relation to burnout, there were no statistical differences between the control and experimental groups.
Burger and Lockhart (2017)	USA	RCT	28 (I)<br>24 (C)	UG Nursing Students	Mindfulness Meditation	10 mins daily over 4 weeks	Attention [44], stress [45] and mindfulness [46]	Meditation demonstrated moderate strength for enhancing executive attention, $F = 4.26$ (1, 49), $n^2 = .080$, $p = .044$. Additional outcomes specific to the meditation group were reduced stress and increased mindfulness, $F = 7.16$ (2,

								47), n^2 = .234, p = .002.
Chen et al. (2013)	China	RCT	30 (I) 30 (C)	UG Nursing Students	Mindfulness Meditation	30min daily for 7 consecutive days	Anxiety [47], depression [48] and autonomic nervous system activity	Differences between pre- and post-treatment Self-Rating Anxiety Scale scores were significantly larger in the meditation group than in the control group, but no similar effect was observed for Self-Rating Depression Scale scores. Systolic blood pressure was reduced more after the intervention in the meditation group than in the control group, with an average reduction of 2.2 mmHg.
Karaca et al. 2019)	Turkey	RCT	190	UG Nursing Students	MBSR	90-95 min sessions twice weekly for 12 weeks	Stress [49] and mindfulness [50]	Statistically significant difference in stress reduction at mean post-test

								and mean follow-up for the control and intervention groups. Statistically significant difference in Mindfulness scores between mean pre-test and post-test obtained by the intervention and control groups.
Koren (2017)	USA	2 x Pilot quasi-experimental design studies	Study 1: 8 (E) and 5 (C) Study 2: 32 (E) and 27 (C)	UG Nursing Students	Mindfulness training	Study 1: 10-minute mindfulness training once a week for six weeks<br><br>Study 2: Five mindfulness interventions for 10 minutes over a period of 11 weeks.	Stress [45] and Awareness [51]	Decreased nursing students' stress and increased awareness, but did not reach significance.

Marthiensen et al. (2019)	Canada	Qualitative	5	UG Nursing Students	Brief MBSR	2 x 4-hour training sessions and 4 weeks of daily practice	N/A	Four themes: (a) hitting a reset button, (b) self-compassion, (c) avoiding a downward spiral, and (d) using an internal coping mechanism.
Niessen and Jacobs (2014)	Netherlands	Mixed Methods Pilot cohort study	123 (I) 78 (WL)	UG Nursing Students	Mindfulness Meditation	4-week intervention	Mindfulness [46] and self-compassion scale [52]	28 students in the intervention group and eight in the control group completed the pre- and post-test questionnaires, findings did have statistical significance. Two qualitative themes: (a) Self-reflection and self-insight (b) Expectations of the training
Ratanasiripong et al. (2015)	USA	RCT	89	UG Nursing Students	Biofeedback and Mindfulness Meditation	Group 1: Biofeedback 3 times per day for 4 weeks	Anxiety [53] and stress [45]	Biofeedback significantly reduced anxiety and maintained stress levels in

						Group 2: Mindfulness Meditation 3 times per day for 4 weeks		nursing students. Mindfulness meditation similarly decreased anxiety levels, while also significantly lowering stress levels. The biofeedback group exhibited significant reduction in anxiety levels among the three groups at post intervention
Sanko et al. (2016)	USA	Time series, two group interventional design	27	UG (n=20) and PG (n=7) Nursing Students	Mindfulness Programme	8 x sixty minute sessions	Mindfulness [54] and Moral Judgement [55]	Improvement in mindfulness scores *p* = 0.003 was found. UG nursing students did not show a statistically significant improvement in mindfulness scores pre to post training (*p* = 0.281), however the post graduate group did (*p* = 0.004).

								Statistically significant pre - post scores were found in moral judgement scores (P [Post conventional] score, *p* = 0.039 and N2 [Maintaining norms] score, *p* = 0.032).
Song and Lindquist (2014)	South Korea	RCT	25 (I) 25 (WL)	UG Nursing Students	MBSR	2h session once weekly for 8 weeks	Depression, anxiety and stress [56] and mindfulness [57]	Compared with WL participants, MBSR participants reported significantly greater decreases in depression, anxiety and stress, and greater increase in mindfulness
Spadaro and Hunker (2016)	USA	Descriptiv e study	26	UG Nursing Students	Online MBSR	1 x session per week for 8 weeks	Stress [45], Anxiety and Depression [58] and cognition [44]	Stress was significantly reduced ($F(2,24) = 4.163$, $p = .019$). A decreasing trend for anxiety was noted with significant difference

								between time points (F(1,23) = 6.889, p = .015) when practice frequency was weekly to daily. Cognition: ability to shift attention, attention selection, concentration, and accuracy improved
Van der Riet et al. (2015)	Australia	Descriptive qualitative design	10	UG Nursing Students	Stress management and mindfulness program	7 x one-hour weekly sessions	N/A	Three main themes emerged: (a) attending to self, (b) attending to others and (c) attending to program related challenges. Data indicated a positive impact on sleep, concentration, clarity of thought and a reduction in negative cognitions. Participants also identified challenges related to timetabling,

								program structure and venue.
VanKuiken et al. (2017)	USA	Descriptive Survey study	151	UG (n=59) and PG (n=92) Nursing Students	Mindfulness meditation and mindful movement strategies	Beginning of class for a full semester	N/A	Four themes emerged: (a) calming/relaxing; (b) focusing/centering; (c) setting aside distractions; and (d) feeling community and connection.
Vilet et al. (2018)	Sweden	Phenomenological hermeneutical qualitative study	26	UG Nursing (n=15) and Medical (n=11) students	Mind-Body skills Course	4 h per week for 5 weeks	N/A	Three main themes were identified: (a) ability to be more present, (b) increased perception and awareness of self, and (c) connection on a deeper level with others.
Yuksel and Yilmaz (2019)	Turkey	Quasi-experimental study	82	UG Nursing Students	Mindfulness-based cognitive therapy	8 x weekly two-hour sessions	Depression, anxiety, stress [59] and mindfulness [50]	Outcomes measured at pre- and post-intervention, and at a 4-months follow-up. The post-test mean scores of Mindful Attention Awareness Scale

	(MAAS) of the experimental group were statistically higher than the control group (p = .006). When the mean scores obtained in the pre-test, post-test and follow-up measurements were compared, the mean scores of MAAS increased (p = .000) and stress scores decreased significantly in the experimental group (p = .004). No statistically significant differences in depression scores.
